# Supplementary material for: In Silico Analysis of Functionalized Hydrocarbon Production Using Ehrlich Pathway and Fatty Acid Derivatives in an Endophytic Fungus
Source: J Fungi (Basel). 2021 May 29;7(6):435. doi: 10.3390/jof7060435 (PMC8228540; doi:10.3390/jof7060435)
Supplement: Supplementary file 1 [file jof-07-00435-s001.zip › 20210525_Supplemental.pdf]

Supplemental material for "*In silico* analysis of functionalized hydrocarbon production using Ehrlich pathway and fatty acid derivatives in an endophytic fungus"

Kristopher A. Hunt, Natasha D. Mallette, Brent M. Peyton, Ross P. Carlson

Culture Medium and Conditions

Ascocoryne sarcoides (NRRL 50072) was grown using 4 ml of media in 25.7 ml sealed test tubes on a minimal cellulose medium consisting of (per liter): KH_2PO_4 (13.6 g), sodium carboxyl methylcellulose (CMC) (10 g), $NaH_2PO_4 \cdot 2H_2O$ (2.75 g), NH_4Cl (1.76 g), $MgSO_4 \cdot 7H_2O$ (0.86 g), $Ca(NO_3)_2 \cdot 4H_2O$ (0.28 g), yeast extract (0.05 g), and trace salts: KCl (60 mg), KNO_3 (80 mg), $FeCl_3$ (2 mg), $MnCl_2$ (5 mg), $ZnSO_4$ (2.5 mg), H_3BO_3 (1.4 mg), and KI (0.7 mg). The initial medium pH was 4.9 and was inoculated with a 7-day culture prepared from Microbank™ Bead frozen stocks (-80°C). Three initial headspace O_2 partial pressures were examined, 21%, 10%, and 7%. 10% and 7% O_2 partial pressures were achieved by purging with N_2 and initial concentrations were confirmed by gas chromatography. O_2 in the liquid accounted for less than 4% of the total present in the vessel. Culture tubes were incubated at 21°C at a fixed 45° angle on rotary shakers at 150 rpm. In addition, each condition was repeated in duplicate as 10 mL volumes in 20 mL solid phase microextraction (SPME) vials.

Biomass was determined in triplicate by cell dry weight (CDW) as described previously [5] and by optical density at 600 nm (OD_{600}). Ammonium was assayed with Hach Method 10031 for High Range Nitrogen (Hach Company, Loveland, CO). CMC, glucose, acetate, and ethanol were measured by high performance liquid chromatography (HPLC) on an Agilent 1200 with an Aminex HPX-87H ion exclusion column at 45°C with 0.005 M H_2SO_4 eluent. O_2 partial pressures were measured with gas chromatography (GC) by 1 mL direct injection of culture tube headspace into a SRI8610C (SRI Instruments, Torrance, CA) with a 6 ft molecular sieve 13x column with helium carrier gas. Volatile organic carbons (VOCs) were measured in the headspace of 20 mL SPME compatible vials (HS-SPME). HS-SPME was performed as described in Mallette et al. 2014 [1].

In vitro functionalized hydrocarbon production

The FHs produced *in vitro* by *A. sarcoides* were a strong function of O_2 partial pressure. The compounds identified in the culture headspace, included alcohols, ketones, aromatics, alkanes, an alkene, an ether, acids, and their esters (Table S1) and ranged from C_2 to C_{15}. Esters represented the greatest number of identified compounds for all three initial conditions. The 7% O_2 partial pressure condition had the most identified compounds, while 21% O_2 partial pressure produced the least. The number of FHs produced at 7 and 10% O_2 partial pressures were similar, despite the observed growth impairment at 7% O_2 partial pressure (Table S1). Quantification of individual compound titers were limited by the HS-SPME technique due to the varied fiber affinity of the compounds in the headspace mixture [5,6] and therefore not reported. However, an integration of the peak areas of the entire mass spectra may approximate the total amount of material adsorbed by the fiber without speciation. The mass of FHs desorbed from the SPME fiber ranged from 123 to 163% higher than abiotic media (i.e. 64 to 82 µg FHs per liter of medium). The highest yield of FHs was at the lowest O_2 condition of 7%, but all yields were at least 10^4-fold lower than the predicted yields that assume maximum conversion indicating the compounds where not likely directly linked to cellular energy production (Figure S1 and S2). The production of reduced carbon byproduct also increased under low O_2 conditions as predicted

by the model; energy generation under increasing O_2 limitation shifted from complete oxidation to byproduct secretion, starting with acetate then ethanol.

Table S1. Observed products of *A. sarcoides* NRRL50072 cultivation under varying initial O_2 partial pressure.

<table>
<tr><th></th><th colspan="3">Initial O_2 partial pressure</th></tr>
<tr><th>Products detected</th><th>7%</th><th>10%</th><th>21%</th></tr>
<tr><td>1,3-octadiene; (Z)-3-octen-1-yl-acetate</td><td></td><td></td><td>2</td></tr>
<tr><td>4-hydroxy-butanoate; (Z)-4-Hexen-1-yl-acetate; (E)-3-decen-2-ol; Furfurylmethylamphetamine; 2-(1-Cyclopent-1-enyl-1-methylethyl) cyclopentanone</td><td></td><td>5</td><td></td></tr>
<tr><td>2-heptanyl-acetate; 3-methyl-butanyl-acetate*; Dimethyl ether; C_2, C_5, and C_{10} alkyl acetate esters*</td><td colspan="2">6</td><td></td></tr>
<tr><td>2-methyl-propanol*; 1,1-dimethyl-cyclohexane; 2-ethyl-hexanol; 2-phenylethanol*; 3-methyl-butyl-2-methyl-butanoate; C_8 alkyl acetate esters*</td><td>6</td><td></td><td></td></tr>
<tr><td>Acetate*; 3-methyl-butanol*; Propyl-cyclopropane*; Octane*; 2-nonanone*; C_7 and C_9 alkyl acetate esters*</td><td colspan="3">7</td></tr>
<tr><td>3-methyl-hexane</td><td>1</td><td></td><td></td></tr>
</table>

*: previously reported [1–5]. The number of products detected under each condition are listed in the shaded boxes.

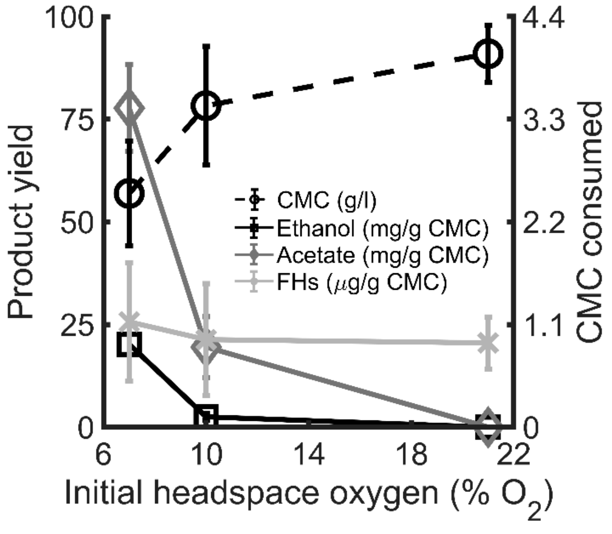

Figure S1. Observed product yields from *A. sarcoides* NRRL 50072 as function of the initial O_2 concentration in the headspace. Error bars represent standard error of measurement for n=6 at stationary phase. Abbreviations: CMC, carboxymethylcellulose; FHs, functionalized hydrocarbons

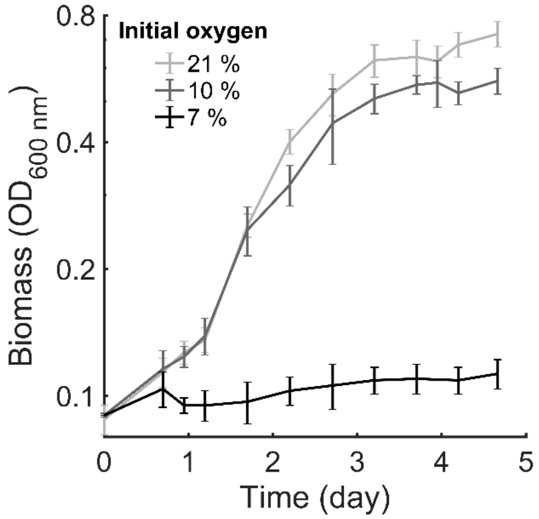

Figure S2. *A. sarcoides* NRRL 50072 biomass production under varying initial O_2 partial pressures. Three initial headspace O_2 partial pressures were examined, 7, 10, and 21%, during biomass production from carboxymethyl cellulose. Values shown are the average and standard error of measurements of biological triplicates.

References

1. Mallette, N.; Pankratz, E.M.; Parker, A.E.; Strobel, G.A.; Busse, S.C.; Carlson, R.P.; Peyton, B.M. Evaluation of cellulose as a substrate for hydrocarbon fuel production by Ascocoryne sarcoides (NRRL 50072). *J. Sustain. Bioenergy Syst.* **2014**, *04*, 33–49, doi:10.4236/jsbs.2014.41004.
2. Strobel, G.A.; Knighton, B.; Kluck, K.; Ren, Y.; Livinghouse, T.; Griffin, M.A.; Spakowicz, D.J.; Sears, J. The production of myco-diesel hydrocarbons and their derivatives by the endophytic fungus *Gliocladium roseum* (NRRL 50072). *Microbiology* **2008**, *154*, 3319–28, doi:10.1099/mic.0.2008/022186-0.
3. Griffin, M.A.; Spakowicz, D.J.; Gianoulis, T.A.; Strobel, S.A. Volatile organic compound production by organisms in the genus *Ascocoryne* and a re-evaluation of myco-diesel production by NRRL 50072. *Microbiology* **2010**, *156*, 3814–29, doi:10.1099/mic.0.041327-0.
4. Gianoulis, T.A.; Griffin, M.A.; Spakowicz, D.J.; Dunican, B.F.; Alpha, C.J.; Sboner, A.; Michael Sismour, A.; Kodira, C.; Egholm, M.; Church, G.M.; et al. Genomic analysis of the hydrocarbon-producing, cellulolytic, endophytic fungus *Ascocoryne sarcoides*. *PLoS Genet.* **2012**, *8*, e1002558, doi:10.1371/journal.pgen.1002558.
5. Mallette, N.D.; Knighton, B.; Strobel, G.A.; Carlson, R.P.; Peyton, B.M. Resolution of volatile fuel compound profiles from *Ascocoryne sarcoides*: a comparison by proton transfer reaction-mass spectrometry and solid phase microextraction gas chromatography-mass spectrometry. *AMB Express* **2012**, *2*, 23, doi:10.1186/2191-0855-2-23.
6. Stashenko, E.E.; Martínez, J.R. Sampling volatile compounds from natural products with headspace/solid-phase micro-extraction. *J. Biochem. Biophys. Methods* **2007**, *70*, 235–242, doi:10.1016/j.jbbm.2006.08.011.
